# The Role of Radiotherapy After Pleurectomy/Decortication for Malignant Pleural Mesothelioma: State of the Art

**DOI:** 10.3390/jpm15120585

**Published:** 2025-12-01

**Authors:** Marco Andolfi, Michele Salati, Majed Refai

**Affiliations:** Thoracic Surgery Unit, AOU (Azienda Ospedaliera Universitaria) of Marche, 60126 Ancona, Italy

**Keywords:** malignant pleural mesothelioma, pleurectomy/decortication, adjuvant therapy, intensity-modulated radiotherapy

## Abstract

**Background**: Considering the increased need to deliver adjuvant radiotherapy (RT) after pleurectomy/decortication (P/D) for malignant pleural mesothelioma (MPM) without exceeding the tolerance of the adjacent normal tissue, new advanced RT technologies have been developed. However, radiation to the whole hemithorax presents a challenge because of the increased risk of toxicity occurring with two intact radiosensitive lungs. The aim of this study is to systematically review the literature in order to assess the role of radiotherapy after P/D for MPM, based on the evidence published so far. **Methods**: We conducted this systematic review according to PRISMA guidelines and registered in an international public register of systematic review (PROSPERO). A PubMed and Cochrane database search was performed to identify articles published from 2005 to 2024 regarding the role of adjuvant radiotherapy after P/D for MPM. We included only level I–III-evidence studies according to the Oxford Centre for Evidence-Based Medicine’s guidance. **Results**: We selected 11 level II studies. Based on published reports, delivery of high-dose external beam ‘conventional’ RT to the entire hemithorax is not recommended in a P/D setting and hemithoracic intensity-modulated radiotherapy (IMRT) may be considered an encouraging and reasonable therapeutic option, leading to excellent loco-regional control and survival results. **Conclusions**: Data and experience strongly support that the ideal platform to define potential indication of the adjuvant RT is a multidisciplinary team. Moreover, given the technical difficulty of IMRT treatment, we recommend considering this treatment in experienced centers with dedicated protocols for MPM due to their ability to detect and manage side effects resulting from the disease and the treatment as well as to ensure the best and the latest treatment plan for each patient.

## 1. Introduction

Malignant pleural mesothelioma (MPM) is a rare, invasive, locally aggressive tumor that is almost always fatal [1]. Despite numerous therapeutic attempts, treatment of MPM represents a major challenge for oncologists, surgeons, and radiation therapists. Indeed, although trimodal therapy, including neoadjuvant chemotherapy followed by surgery and radiotherapy, seems to be the best treatment, the ideal combination of adopted therapies is still an object of debate. Specifically, the role of surgery in the management of MPM is still a matter of controversy [2,3,4] and the choice of which of the different procedures to adopt depends not only on the tumor’s histology, its distribution, and the patient’s pulmonary reserve but also on the surgeon’s experience.

To date lung-sparing surgery with pleurectomy/decortication (P/D) is considered the preferred surgical treatment option and can be considered in select patients for complete gross cytoreduction, guaranteeing 30-day mortality, 90-day mortality, median disease-free, and overall survival comparable to extrapleural pneumonectomy (EPP) [4]. Moreover, P/D is a well-tolerated procedure, allowing for a complete trimodal treatment in most MPM patients (74% P/D group versus 32% EPP group, *p* < 0.001) [4].

Based on these findings, radiation to the whole hemithorax after lung-sparing surgery becomes challenging considering the increased risk of toxicity occurring with two intact radiosensitive lungs [5]. Furthermore, the National Comprehensive Cancer Network (NCCN) Clinical Practice Guidelines remain equivocal on the role of adjuvant radiotherapy (RT) after P/D [6], suggesting consideration of an intensity-modulated radiotherapy (IMRT) approach only in centers with experience and expertise in these methods.

Relatedly, many authors [7,8,9,10,11,12,13,14,15,16,17] have reported different techniques of adjuvant radiotherapy, demonstrating that, in patients with non-metastatic MPM who have undergone lung-sparing surgery, IMRT guaranteed substantially greater overall survival compared to palliative radiotherapy (RT). The aim of this study is to perform a systematic review on the role of radiotherapy after P/D for MPM based on the evidence published so far.

## 2. Methods

We conducted this systematic review according to PRISMA guidelines [18] (PRISMA checklist: Supplementary Materials) and registered in an international public register of systematic review (PROSPERO, ID: 1104284). A PubMed and Cochrane database search was performed to identify articles published from 2005 to 2024 regarding the role of adjuvant radiotherapy after P/D for MPM. We used the following terms for the search: [(malignant pleural mesothelioma OR mesothelioma) AND (pleurectomy/decortication OR pleurectomy OR decortication) AND (adjuvant radiotherapy OR radiotherapy)].

We included level I–III-evidence studies according to the Oxford Centre for Evidence-Based Medicine’s guidance.

Exclusion criteria were level IV–V-evidence studies, guidelines, review articles, videos, content unrelated to the topic, articles not written in English, and studies edited prior to 2005.

**Figure 1:**
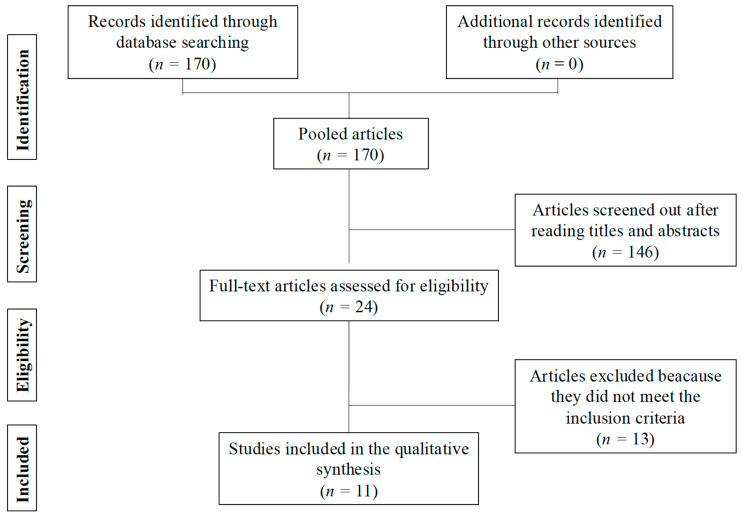
Diagram illustrating study selection according to PRISMA guidelines.

One hundred and seventy papers were found using the reported search (see Figure 1). Once duplicates were removed, items were screened out after through reading titles and abstracts in order to evaluate the accuracy of the search and select only those specifically related to the topic. Thereafter, articles without specific evaluable clinical outcomes or not meeting the aim of the study were excluded. In total, 146 remaining eligible articles were removed, leaving a total of 24 publications assessed for eligibility. 

The manuscripts which we considered to potentially describe adjuvant radiotherapy after P/D were assessed in their full texts. Additional studies were investigated through a manual search of the references of the selected articles. Of these 24 articles, 13 were excluded because they did not contain the aforementioned parameters and 11 were included in the qualitative synthesis.

The selection procedure of this systemic review is shown in Figure 1.

The authors extracted complete data on patient groups and outcomes (locoregional control—LRC; overall survival—OS; progression free survival—PFS; toxicity) from each study.

Systematic searches were independently performed by two authors (AM and SM).

The main adjuvant radiotherapy techniques are discussed in detail in Section 3.

## 3. Results

No level I or III studies focusing on the selected topic were found in the study period. Of the 11 level II studies included in the qualitative synthesis [7,8,9,10,11,12,13,14,15,16,17], 8 level II studies examined [7,10,11,12,14,15,16,17] the role of radical IMRT as an adjuvant RT in patients who had undergone P/D and neoadjuvant chemotherapy for MPM, demonstrating better outcomes (LRC and OS) compared to those reported for older techniques [8,9] especially when delivered using conventionally fractionated regimens (45 to 54 Gy). Moreover, the incidence of grade ≥ 2 pneumonitis after IMRT ranged from 2% to 30% (mean 17.6%). Among these studies, only one [11] reported fatal pneumonitis 3 months after treatment correlated with dosimetric parameters. Median overall survival ranged from 19 to 33 months (mean 23.6).

One level II study [13] described proton and photodynamic therapy, reporting excellent results in terms of LRC and OS without recording grade ≥ 3 acute or late toxicities, and 2 level II studies [8,9] evaluated the impact of high-dose external beam ‘conventional’ RT to the entire hemithorax, showing that it was not recommended in a P/D setting as it resulted in significant toxicity.

However, this systematic review has some bias mainly due to the rarity of the disease (hence the limited sample size), the consequent inhomogeneity of the surgical procedures described, the different chemotherapy regimens adopted, and the absence of comparative studies able to define the effective impact of the adjuvant RT.

The main characteristics of the 11 studies included in the systematic review are detailed in Table 1.

Specifically, Franceschini et al. [7] performed a retrospective cohort analysis on the use of adjuvant volumetric modulated arc therapy (VMAT) in 49 patients with MPM who underwent P/D. Of these, 47 patients (96%) had been treated with neoadjuvant chemotherapy. With a median follow-up period of 27.4 months, the median duration of LRC was 53.7 months, the median PFS was 14.9 months, and the median OS was 21.5 months.

In addition, the study showed that VMAT treatment was well-tolerated and severe toxicity was reduced when compared with historical data obtained on older techniques, showing only two grade 3 acute toxicities, one grade 5, and two grade 4 toxicities during the follow-up. However, results were still unsatisfactory considering that high rates of local and distant failure and bleak prognoses for most patients were recorded and that the heart doses > 20 Gy and >30 were related to the development of pulmonary toxicity (*p* = 0.018 and 0.077, respectively).

Shaik et al. [8] demonstrated that, in 209 patients who had undergone P/D and adjuvant RT, OS was significantly higher in patients treated with IMRT compared with those receiving conventional external beam RT (median 20.2 months vs. 12.3 months, respectively; *p* = 0.001). No significant differences in the cumulative incidence of local failure progression between the groups were recorded. However, the IMRT patient group developed fewer instances of grade ≥ 2 esophagitis compared with the conventional group.

Gupta et al. [9] conducted a retrospective cohort study enrolling 123 patients who had undergone P/D and adjuvant external beam RT. Only 14 patients (11%) received chemotherapy (6 neoadjuvant and 8 adjuvant). The authors concluded that P/D with adjuvant RT is not an effective treatment option for patients with MPM, showing a median OS of 13.5 months and a 2-year actuarial OS rate of 23%. Moreover, two patients (1.6%) died with Grade 5 toxicity within 1 month of radiotherapy.

Furthermore, the authors demonstrated that for improving LRC and OS, residual disease might require a more extensive surgery followed by external RT rather than an external RT with or without brachytherapy.

Rimner et al. [10] conducted a two-center phase II study to determine the safety of hemithoracic IMRT (50.4 Gy with no integrated boost) in 27 patients with MPM. Their results showed that hemithoracic IMRT was safe, demonstrating an acceptable rate of radiation pneumonitis (no grade 4 or 5 radiation-related toxicities were observed). Moreover, the 1- and 2-year OS rates for patients with resectable disease were 80% and 59%, respectively, concluding that IMRT with neoadjuvant chemotherapy and P/D has the potential to be a new lung-sparing treatment paradigm for locally advanced MPM.

Minatel et al. [11] conducted a prospective study to evaluate the OS, PFS, toxicity and prognostic factors in 69 patients who had undergone IMRT (50 Gy + 10 Gy integrated boost in 3 patients) after P/D. Comparing two groups (n = 35 extended P/D; n = 34 partial P/D), they showed that OS, PFS, and LRC were not different, and the radiation toxicity was acceptable, recording 14 cases (20%) of grade 2 to 3 pneumonitis. However, they recorded one fatal case of pneumonitis 3 months after treatment correlated with dosimetric parameters (total lung V20, 45%; total lung V30, 1%; mean lung dose, 22 Gy). Indeed, they demonstrated that total lung V20 and V30 and mean lung dose were correlated with the risk of severe radiation pneumonitis (RP, *p* = 0.04). However, local failure rates were low (19%) and 2-year OS was high (58–65%).

Rimner et al. [12] reported a detailed pattern-of-failure analysis and OS in 67 patients with MPM who were unresectable or had undergone IMRT after P/D and neoadjuvant chemotherapy (85% of the entire cohort). Their study showed that 43 patients (64%) experienced in-field local failure with a 1- and 2-year actuarial failure rate of 56% and 74%, respectively.

Furthermore, when comparing the two groups (P/D versus partial or unresectable pleurectomy), it was found that the 1- and 2- year in-field failure rates were 43% and 60% vs. 66% and 83%, respectively (*p* = 0.03). Similarly, the 1- and 2-year actuarial OS rates were 89% and 57% vs. 82% and 42% (the median OS was 26 months vs. 22 months).

Rice et al. [13] conducted the largest prospective trial of proton therapy (PT) for MPM to date, enrolling 10 consecutive patients treated with PT as an adjuvant therapy following P/D and neoadjuvant chemotherapy (7/10) in order to evaluate the toxicities, LRC, and OS rates.

The results showed excellent local control (90%), as well as excellent OS rates of 56% at 1 year and 29% at 2 years for patients undergoing P/D with photodynamic therapy followed by PT. Furthermore, this is the only study that reported no grade ≥ 3 acute or late toxicities.

Minatel et al. [14] conducted a prospective study to evaluate the OS, PFS, and LRC in 20 patients who had undergone high doses of RT (helical tomotherapy) after radical P/D. Nineteen patients (95%) received chemotherapy (11 adjuvant and 8 both neoadjuvant and adjuvant).

Specifically, they demonstrated that the high doses of RT delivered after radical P/D led to excellent LRC and OS results in the entire cohort of study, showing a median OS and PFS of 33 and 29 months and a 3-year OS and PFS rate of 49% and 46%, respectively, with the predominant pattern of failure being a distant occurrence.

Parisi et al. [15] conducted a retrospective cohort study reporting 36 patients with MPM who had undergone IMRT after P/D (53%) and after pleural biopsy (47%), delivering the lowest median dose radiation compared with the other studies included in this review (25 Gy). With regard to patients who had undergone P/D followed by RT, the 1- and 2-year OS rates were 85% and 40%, respectively, and a median survival of 21.6 months [95% confidence interval (95% CI): 15.5–24.1] compared to 19.4 months for patients who were deemed to be unresectable. Moreover, three patients developed grade 3 pneumonitis, five had grade 2 dyspnea, and six had grade 2 cough.

Harrabi et al. [16] evaluated the toxicity, PFS, and the OS in 10 MPM patients who had undergone IMRT after P/D. All patients received chemotherapy (2 neoadjuvant and 8 adjuvant).

Their study showed a reasonable toxicity profile concerning the remaining intact lung, considering that no treatment-related toxicity exceeding grade 3 was recorded. Furthermore, they reported a median PFS and OS of 13 and 19 months, respectively.

Finally, Arrieta et al. [17] performed a prospective study, enrolling 15 patients who had undergone trimodal therapy (platinum-based chemotherapy followed by P/D and IMRT). They showed an acceptable locoregional control in patients with locally advanced disease (LRC at two years was 75.9%), a median OS of 23.6 months, and a median PFS of 18.9 months. Furthermore, concerning the toxicity of the radiation treatment, nine patients (60%) were recorded to have pneumonitis, and, of these, two patients (13.3%) experienced grade 3 or 4 radiation pneumonitis.

## 4. Discussion

With the spread of lung-sparing surgery favored over EPP for patients with MPM, the need to deliver adjuvant RT is increased, considering the decreased probability of achieving complete surgical resection.

Based on the papers published to date [7,8,9,10,11,12,13,14,15,16,17], radical IMRT seems to be the most appropriate approach in patients who have undergone P/D and neoadjuvant chemotherapy for MPM, demonstrating a better treatment toxicity profile and a higher median of OS rates compared to those reported in data on older techniques [8,9].

Indeed, as reported by Shaik and Gupta [8,9], delivery of high-dose external beam ‘conventional’ RT to the entire hemithorax was not recommended in a P/D setting, as it did not show a significant clinical benefit and it was proven to result in significant toxicity. Therefore, due to the need to irradiate a large target volume without exceeding the tolerance of the adjacent normal tissue, especially the ipsilateral intact lung, new advanced RT technologies were developed [7,10,11,12,13,14,15,16,17]. Specifically, this systematic review demonstrated that hemithoracic IMRT after lung-sparing surgery and neoadjuvant chemotherapy has the potential to be a new acceptable and concrete treatment paradigm for MPM, reducing areas of dose uncertainty and doses to underlying organs at risk. Moreover, clinical outcomes (median OS and PFS) of patients who have undergone IMRT after P/D were comparable to those reported with the implementation of trimodal regimens using EPP [4,19,20]. These data confirmed results reported in the systematic review conducted by Patel et al. [21] regarding toxicities and disease-related outcomes in patients with MPM after adjuvant IMRT. Indeed, they demonstrated that this approach was safe and could be considered in well-selected patients, producing few higher-grade toxicities and showing a reasonable disease-control outcome, especially when a dose of 45–54 Gy was delivered.

Moreover, de Perrot demonstrated in a review including 14 studies how this radiotherapy approach could potentially have an intrinsic benefit even after EPP, guaranteeing a promising median survival of patients who completed the treatment with acceptable toxicity rates [22]. However, as reported by Mosleh et al., when comparing overall survival of patients who have undergone therapies including neoadjuvant versus adjuvant IMRT, no relevant benefits were revealed (median OS 17.5 vs. 24.0 months, *p* = 0.39) [23].

Other radiation techniques after lung-sparing surgery have been described [7,24,25,26,27]. In this context, VMAT and intensity-modulated helical tomotherapy (IMHT) showed promising results, resulting in satisfactory local control and survival with acceptable toxicities. Specifically, Ozyurt et al. demonstrated the efficacy and tolerability of IMHT in 11 patients with MPM who received trimodal therapy, even if they found a significant association between RP and medulla spinalis and esophageal doses (*p* < 0.05) [25]. Instead, Parisi reported the preliminary toxicity results of the first 20 patients treated with accelerated hypofractionated radiotherapy, showing that it was safe and feasible and recording only one case of G3 toxicity (RP) [26].

However, because of the rarity of this disease, controversy regarding the indication of surgical resection and the inhomogeneity of the procedures described, the role of RT after lung-sparing surgery remains controversial and an individualized patient-oriented decision is recommended [6,28,29]. In addition, in the only comparative study between patients receiving adjuvant radiation or no radiation after definitive surgery for pathologic stage I-III MPM, the authors showed that adjuvant treatment was not associated with improved OS and did not reduce the incidence of recurrence [30].

Moreover, many studies grouped outcomes of IMRT with other patients who were non-operable or underwent only a palliative procedure [10,12,15]. The use of an integrated boost was nonuniformly performed and the timing of chemotherapy was not standardized. Finally, these data were reported by high-volume centers with experience treating MPM; therefore, the results of this review may not be applicable to patients treated in small centers.

In light of these limitations, data and experience strongly support that the ideal platform to define the potential indication of adjuvant RT is a multidisciplinary team composed of surgeons, radiation oncologists, radiologists, medical oncologists, and pulmonologists with experience in managing pleural mesothelioma. Indeed, given the technical difficulty of IMRT treatment, we recommend considering this treatment in experienced centers with dedicated protocols for MPM diagnosis and treatment until such time as a standardized clinical practice can be consolidated by prospective comparative trials [8,10,11].

In particular, based on the published studies, tertiary referral centers with mesothelioma programs and specialized multidisciplinary teams are suggested for starting these protocols of care due to their ability to detect and manage side effects resulting from the disease and the treatment as well as to ensure the best and the latest treatment plan for each patient.

In conclusion, to the best of our knowledge, this is the first systematic review focusing on the role of different adjuvant RT approaches after P/D, and it has demonstrated that the delivery of high-dose external beam ‘conventional’ RT to the entire hemithorax is not recommended in a P/D setting and hemithoracic IMRT may be considered an encouraging and reasonable therapeutic option for MPM, leading to excellent loco-regional control and survival results.

More and larger studies, including comparative evaluations of different techniques of adjuvant RT, are needed to confirm the effectiveness of IMRT and its real clinical impact in patients with MPM who have undergone P/D. Moreover, larger cohorts of patients, adequate monitoring of pulmonary and cardiac toxicities as well as the quality of life of patients, and comparative studies regarding adjuvant radiotherapy with protons versus IMRT could help to define the role of adjuvant radiotherapy.

## Figures and Tables

**Table 1:** Studies included in the systematic review.

Author, Date, Journal and Country, Study Type (Level of Evidence)	Patient Group	Outcomes	Key Results	Dose Radiation	Comments
Franceschini et al. (2020) [7] Clin. Lung Cancer, Italy (level II)	49 patients underwent P/D and adjuvant RT with VMAT	LRC (2, 24, 36 months)	75.2%, 67.4%, 56.5%.	44 Gy (22–59.4 Gy)	Limited sample size.
		PFS (months)	14.9		Carboplatin- instead of cisplatin-based chemotherapy and R2 resection showed a negative correlation with OS.
		OS (months)	21.5		The percentage of the heart receiving >20 Gy and >30 was associated with late pneumonitis.
		Toxicity (Grade ≥ 2 fatigue, lung fibrosis, RP, dyspnea)	6.2%, 4.1%, 26.6%, 8.2%		A total of 47 patients (96%) treated with neoadjuvant chemotherapy.
Shaik et al. (2017) [8] J. Thorac. Oncol. USA (level II)	209 patients underwent P/D and adjuvant RT (Group A[CONV] = 131, Group B[IMRT] = 78)	OS (months)	A: 12.3 B: 20.2 (*p* = 0.001)	[CONV]: >45 Gy: 11% pts [IMRT] >45 Gy: 65% pts	Long enrolment period (>40 years).
		LRC (1- and 2-year rates)	A: 34%, 47% B: 42%, 60% (*p* = 0.08)		Association between adjuvant hemithoracic IMPRINT, chemotherapy, and P/D with promising OS rates and decreased toxicities.
		PFS (1- and 2-year rates)	A: 47%, 69% B: 53%, 72% (*p* = 0.07)		84 patients (41%) received chemotherapy (11% in group A and 90% in group B).
		Toxicity (Grade ≥ 2 esophagitis, fatigue, cough)	A: 47%, 16%, 2% B: 23%, 47%, 18%		No significant difference in grade 3/4 RP, nausea, vomiting, dyspnea and dermatitis
Gupta et al. (2005) [9] Int. J. Radiat. Oncol. Biol. Phys. USA (level II)	123 patients underwent P/D and adjuvant external beam RT	OS (months)	13.5	42.5 Gy (7.2–67.8 Gy)	Radiation dose <40 Gy, nonepithelioid histology, left-sided disease, and use of an implant are unfavorable for OS. 14 patients (11%) received chemotherapy (6 neoadjuvant and 8 adjuvant).
		(2-year rate)	23%		
		LRC (1-year)	42%		
		Toxicity (Grade ≥ 2 esophagitis, fatigue, RP, dyspnea)	48.7%, 11.3%, 35.7%, 13.8%		
Rimner et al. (2016) [10] J. Clin. Oncol. USA (level II)	27 patients underwent IMRT for MPM (Group A = 11 unresectable; Group B = 16 P/D and neoadjuvant chemotherapy)	Toxicity (Grade ≥ 2 RP, esophagitis, fatigue, dyspnea)	29.6%, 29.6%, 40.7%, 44.4%	46.8 Gy (28.8–50.4 Gy)	Limited sample size.
		PFS (months)	12.4		
		OS (months)	23.7		
		(1- and 2-year rates)	A: 74%, 25% B: 80%, 59%		
Minatel et al. (2015) [11] Int. J. Radiat. Oncol. Biol. Phys. Italy (level II)	69 patients underwent IMRT after P/D (Group A = 35 extended P/D; Group B = 34 partial P/D)	OS (2–3 year)	A: 65%, 44% B: 58%, 36% (*p* = 0.94).	50 Gy	Surgery elsewhere. Patients with immediate progression after surgery or chemotherapy were not enrolled. 19 patients (27.5%) developed distant metastases. Correlation between gross residual disease after surgery and OS. 69 patients (100%) received chemotherapy (8 neoadjuvant, 53 adjuvant, and 8 both).
		LRC (2–3 year)	A: 65%, 58% B: 64%, 42% (*p* = 0.75).		
		PFS (2–3 year)	A: 50%, 40% B: 40%, 38% (*p* = 0.76).		
		Toxicity (Grade ≥ 2 RP)	20%		
Rimner et al. (2014) [12] Int. J. Radiat. Oncol. Biol. Phys. USA (level II)	67 patients underwent IMRT after P/D (Group A = 28 extended P/D; Group B = 39 partial P/D or unresectable)	LRC (1–2 year in field local failure)	56%, 74% A: 43%, 60% B: 66%, 83% (*p* = 0.03)	46.8 Gy (45–50.4 Gy)	43 patients (64%) experienced in-field local failure; 13 (19%) a marginal failure; 25 (37%) had out-of-field failure, and 32 patients (48%) had distant failure. 57 patients (85%) received neoadjuvant chemotherapy. No data regarding acute and late toxicities was reported.
		(1–2 year distant failure)	40%, 55%		
		OS (months)	24		
		(1–2 year rates)	85%, 50%		
Rice et al. (2019) [13] Photochem. Photobiol. USA (level II)	10 patients underwent chemotherapy, P/D/proton therapy and photodynamic therapy	LRC (1–2 year rates)	90%, 90%	55 CGE (50–75 CGE)	Limited sample size. 70% of patients received neoadjuvant chemotherapy.
		OS (months)	30.3		
		(1–2 year rates)	58%, 29%		
		Toxicity (Grade 2 RP, cough, dyspnea, dysphagia)	10%, 10%, 20%, 10%		
Minatel et al. (2014) [14] Lung Cancer Italy (level II)	20 patients underwent P/D and adjuvant RT	OS (months)	33	46 Gy	Surgery elsewhere. 7 patients (35%) had distant failure; 3 patients (15%) had an isolated loco-regional recurrence. 19 patients (95%) received chemotherapy (11 adjuvant and 8 both neoadjuvant and adjuvant).
		(1–3 year rates)	70%, 49%		
		PFS (months)	29		
		(2–3 year rates)	65%, 46%		
		LRC (2–3 year rates)	68%, 59%		
		Toxicity (Grade ≥ 2 RP, pericardial effusion)	25%, 10%		
Parisi et al. (2017) [15] Cancer/Radiothérapie Italy (level II)	36 patients received IMRT (Group A = 19 P/D; Group B = 17 biopsy)	Toxicity (Grade ≥ 2 RP, dyspnea, cough)	9%, 14%, 17%	25 Gy (25–30 Gy)	29 patients (80%) received chemotherapy (no data regarding the timing).
		OS (months)	21.6		
		(1–2 year rates)	A: 85%, 40%		
Harrabi et al. (2017) [16] Rep. Pract. Oncol. Radiother. Germany (level II)	10 patients underwent P/D and adjuvant IMRT	Toxicity (Grade ≥ 2 RP)	20%	52.2 Gy (40–54 Gy)	Limited sample size. 10 patients (100%) received chemotherapy (2 neoadjuvant and 8 adjuvant).
		PFS (months)	13		
		OS (months)	19		
Arrieta et al. (2020) [17] Thorac. Cancer Mexico (level II)	15 patients underwent trimodal therapy (chemotherapy, P/D, and adjuvant IMRT)	Toxicity (Grade ≥ 3 RP, esophagitis, fatigue)	2%, 2%, 3%	48.7 Gy (23.4–54 Gy)	Limited sample size. Pulmonary function tests were not performed
		PFS (months)	18.9		
		OS (months)	23.6		
		LRC (2year rate)	75.9%		

RT: radiation therapy; VMAT: volumetric modulated arc therapy; RP: radiation pneumonitis; P/D: pleurectomy/decortication; IMRT: intensity modulated RT; MPM: malignant pleural mesothelioma; OS: overall survival; PFS: progression free survival; SIB: simultaneous integrated boost; LRC: locoregional control.

## Data Availability

No new data were created or analyzed in this study. Data sharing is not applicable to this article.

## Supplementary Materials

The following supporting information can be downloaded at: https://www.mdpi.com/article/10.3390/jpm15120585/s1, Table S1: PRISMA checklist.
